# Supplementary material for: On the evolutionary ecology of multidrug resistance in bacteria
Source: PLoS Pathog. 2019 May 13;15(5):e1007763. doi: 10.1371/journal.ppat.1007763 (PMC6532944; doi:10.1371/journal.ppat.1007763)
Supplement: S1 Text — PDF file containing all supporting text and figures. (PDF) [file ppat.1007763.s001.pdf]

On the evolutionary ecology of multidrug resistance in bacteria: Supporting Information

The Maela Pneumococcal Collaboration

April 26, 2019

1 Implicit modelling of balancing selection

In the main text, we show that for the purposes of understanding association between resistance determinants, models of competition between antibiotic resistance and sensitivity with host population structure and strain structure can both be simplified to a series of independent SIS models. In the case of strain structure, this requires the presence of balancing selection to maintain the strains differing in duration of carriage. We do not model this balancing selection explicitly: we assume coexistence of the strains and focus on modelling the competition between sensitivity and resistance within each strain.

To illustrate this simplification, we consider a model with n strains in which strain coexistence is maintained by scaling the transmission rate of strain i (β_i) by $g(f_i)$, where f_i is the frequency of strain i and $g()$ is a decreasing function (this is the model in Lehtinen et al. [1]). For any strain i, with sensitive and resistant sub-strains is and ir, clearance rate μ_i, cost of resistance c_β ($c_\beta \leq 1$) and c_μ ($c_\mu \leq 1$) and population antibiotic consumption τ, the competition between sensitivity and resistance is captured by:

$$\begin{aligned} \frac{dI_{is}}{dt} &= \beta_i I_{is} g(f_i) U - (\tau + \mu_i) I_{is} \\ \frac{dI_{ir}}{dt} &= c_\beta \beta_i I_{ir} g(f_i) U - \frac{\mu_i}{c_\mu} I_{ir} \end{aligned} \tag{1}$$

This model predicts competitive exclusion within strain: when $c_\beta c_\mu (1 + \frac{\tau}{\mu_i}) > 1$, strain i will be resistant ($I_{is} = 0$, $I_{ir} > 0$) and when $c_\beta c_\mu (1 + \frac{\tau}{\mu_i}) < 1$, strain i will be sensitive ($I_{is} > 0$, $I_{ir} = 0$). Importantly, these expression depend only on strain i: the competition between sensitivity and resistance within strain i is independent of the other strains in the model. For our purposes, therefore, we can model each strain separately, replacing $g(f_i)U$ by a fixed number of hosts available to strain i, giving rise to the model presented in the main text.

2 Schematics of resistance profiles

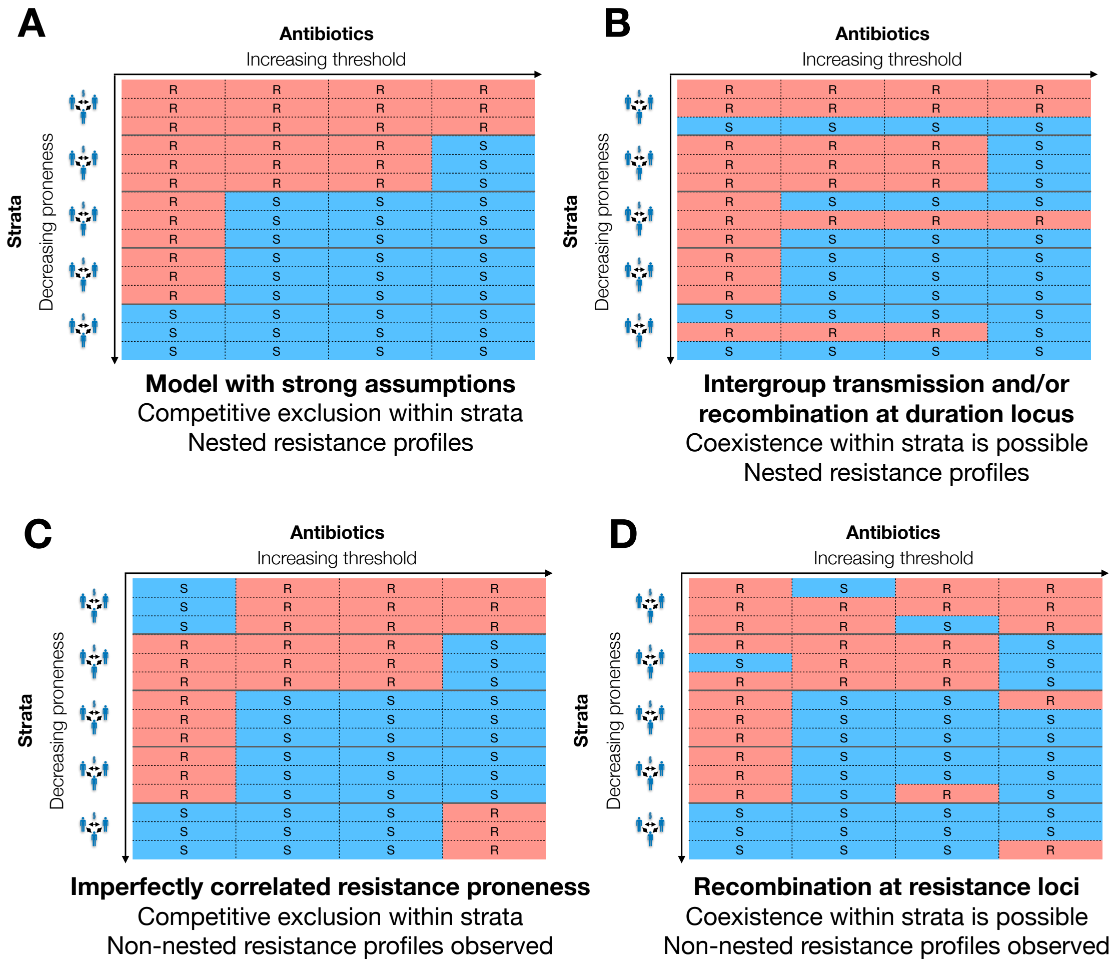

Figure A: Schematic illustrating the effect of relaxing various model assumption on a set of resistance profiles from a system with five strata and four antibiotics. A: set of resistance profiles under the model with strong assumptions (main text Figure 2). B: Mixing between strata (integroup transmission or recomination at the duration locus) introduces some within-strata diversity in resistance profiles, but all observed resistance profiles have a nested structure. C: If the resistance proneness of a strata is not the same for all antibiotics, non-nested resistance profiles can arise. D: Recombination at the resistance loci breaks down associations between resistances and leads to the presence of non-nested resistance profiles and coexistence of multiple resistance profiles within strata.

3 Effect of resistance on clearance rate in multidrug model

In Sections 2.1.3 and 2.1.4 of the main text, when considering selection for resistance against antibiotic a, we formulate the model in a way that ignores the effect of treatment with, and resistance against, *other* antibiotics. Resistance against other antibiotics affects clearance rate: firstly, by determining whether the strain can be cleared through exposure to the other antibiotics and, secondly, through the effect of fitness cost of resistance (if applicable). Therefore, the absence/presence of resistance against other antibiotics will affect selection for resistance to antibiotic a.

To investigate the impact this effect has on our predictions about the association between resistance determinants, we consider a system with two antibiotics and competition between four resistance profiles: sensitive to both antibiotics (SS); sensitive to antibiotic 1, resistant to antibiotic 2 (SR); resistant to antibiotic 1, sensitive to antibiotic 2 (RS); resistant to both (RR). Within a particular stratum with total antibiotic consumption τ and clearance rate μ, the dynamics of these strains are captured by:

$$
\begin{aligned}
\frac{dI_{SS}}{dt} &= \beta I_{SS}U - (\tau + \mu)I_{SS} \\
\frac{dI_{SR}}{dt} &= c_{\beta 2}\beta I_{SR}U - (\gamma_1\tau + \frac{\mu}{c_{\mu 2}})I_{SR} \\
\frac{dI_{RS}}{dt} &= c_{\beta 1}\beta I_{RS}U - (\gamma_2\tau + \frac{\mu}{c_{\mu 1}})I_{RS} \\
\frac{dI_{RR}}{dt} &= c_{\beta 1}c_{\beta 2}\beta I_{RR}U - \frac{\mu}{c_{\mu 1}c_{\mu 2}}I_{RR}
\end{aligned}
\tag{2}
$$

where $U + I_{SS} + I_{SR} + I_{RS} + I_{RR} = 1$; β is the transmission rate; $c_{\beta 1} \leq 1$ and $c_{\beta 2} \leq 1$ are the cost of resistance to antibiotic 1 and 2 respectively on transmission and $c_{\mu 1} \leq 1$ and $c_{\mu 2} \leq 1$ are the costs on clearance; and γ_1 and γ_2 are the proportion of total antibiotic consumption corresponding to each antibiotic. Without loss of generality, we assume antibiotic 2 is prescribed at a higher rate than antibiotic 1 ($0.5 > \gamma_1 = 1 - \gamma_2$).

This system will give rise to incomplete linkage disequilibrium ($D' < 1$) between the resistances (and non-nested resistance profiles) if there are strata (i.e. some values of resistance proneness $\frac{\tau}{\mu}$) where strain RS is selected for and other strata (i.e. other values of $\frac{\tau}{\mu}$) where strain SR is selected for. The basic reproductive number ($R0$) of RS is $\frac{c_{\beta 1}\beta}{\frac{\mu}{c_{\mu 1}}+\gamma_2\tau}$ and the basic reproductive number of SR is $\frac{c_{\beta 2}\beta}{\frac{\mu}{c_{\mu 2}}+\gamma_1\tau}$. SR out-competes RS when $R0_{SR} > R0_{RS}$. Re-arranging this gives:

$$\frac{\tau}{\mu} > \frac{c_{\beta 1}c_{\mu 1} - c_{\beta 2}c_{\mu 2}}{c_{\mu 1}c_{\mu 2}(\gamma_2 c_{\beta 2} - \gamma_1 c_{\beta 1})}$$

When the fitness cost of the more commonly prescribed antibiotic (antibiotic 2) is lower ($c_{\beta 1}c_{\mu 1} < c_{\beta 2}c_{\mu 2}$, $c_{\beta 1} \leq c_{\beta 2}$ and $c_{\mu 1} \leq c_{\mu 2}$), the inequality will hold independent of the value of μ and τ (as long as $u > 0$ and $\tau \geq 0$): the left-hand side is always positive, whereas the right-hand side is always negative. Thus, resistance to the more commonly prescribed antibiotic with lower fitness cost is always more beneficial than resistance to the other drug and SR is fitter than RS in all strata.

When the fitness cost of the more commonly prescribed antibiotics is greater ($c_{\beta 1}c_{\mu 1} > c_{\beta 2}c_{\mu 2}$, $c_{\beta 1} \geq c_{\beta 2}$ and $c_{\mu 1} \geq c_{\mu 2}$), the relative fitness of SR and RS depends on the antibiotic consumption rate and clearance rate and may therefore differ between strata. However, incomplete linkage disequilibrium will only arise if a change in $\frac{\tau}{\mu}$ leads from a stable equilibrium with SR at fixation to a stable equilibrium with RS at fixation (or, equivalently, vice versa).

We perform a stability analysis to determine the parameter space in which this transition occurs. In line with the result above, stability of the SR equilibrium requires:

$$\frac{\tau}{\mu} > \frac{c_{\beta 1}c_{\mu 1} - c_{\beta 2}c_{\mu 2}}{c_{\mu 1}c_{\mu 2}(\gamma_2 c_{\beta 2} - \gamma_1 c_{\beta 1})}$$

In addition, the following conditions must also be met:

$$
\begin{aligned}
\frac{\tau}{\mu} &< \frac{1 - c_{\mu 1}c_{\beta 1}}{\gamma_1 c_{\mu 1}c_{\mu 2}c_{\beta 1}} \\
\frac{\tau}{\mu} &> \frac{1 - c_{\mu 2}c_{\beta 2}}{c_{\mu 2}(c_{\beta 2} - \gamma_1)}
\end{aligned}
$$

These two conditions are also required for the equilibrium with RS at fixation to be stable, in addition to:

$$\frac{\tau}{\mu} < \frac{c_{\beta 1}c_{\mu 1} - c_{\beta 2}c_{\mu 2}}{c_{\mu 1}c_{\mu 2}(\gamma_2 c_{\beta 2} - \gamma_1 c_{\beta 1})}$$

Therefore, the relative treatment rate (γ_1) and fitness cost parameters at which incomplete linkage disequilibrium may occur are constrained by: $\frac{1-c_{\mu 2}c_{\beta 2}}{c_{\mu 2}(c_{\beta 2}-\gamma_1)} < \frac{c_{\beta 1}c_{\mu 1}-c_{\beta 2}c_{\mu 2}}{c_{\mu 1}c_{\mu 2}(\gamma_2 c_{\beta 2}-\gamma_1 c_{\beta 1})} < \frac{1-c_{\mu 1}c_{\beta 1}}{\gamma_1 c_{\mu 1}c_{\mu 2}c_{\beta 1}}$. In addition, for incomplete linkage equilibrium to actually be observed, there must be at least one stratum with resistance proneness $\frac{\tau}{\mu} > \frac{c_{\beta 1}c_{\mu 1}-c_{\beta 2}c_{\mu 2}}{c_{\mu 1}c_{\mu 2}(\gamma_2 c_{\beta 2}-\gamma_1 c_{\beta 1})}$ and one stratum with resistance proneness $\frac{\tau}{\mu} < \frac{c_{\beta 1}c_{\mu 1}-c_{\beta 2}c_{\mu 2}}{c_{\mu 1}c_{\mu 2}(\gamma_2 c_{\beta 2}-\gamma_1 c_{\beta 1})}$.

This places considerable restrictions on the parameter range at which incomplete linkage equilibrium could be observed. For example, consider a system in which strata correspond to strains differing in clearance rate, with a uniform total antibiotic consumption rate across the strata. For parameter values $c_{\mu 1} = 0.99$,

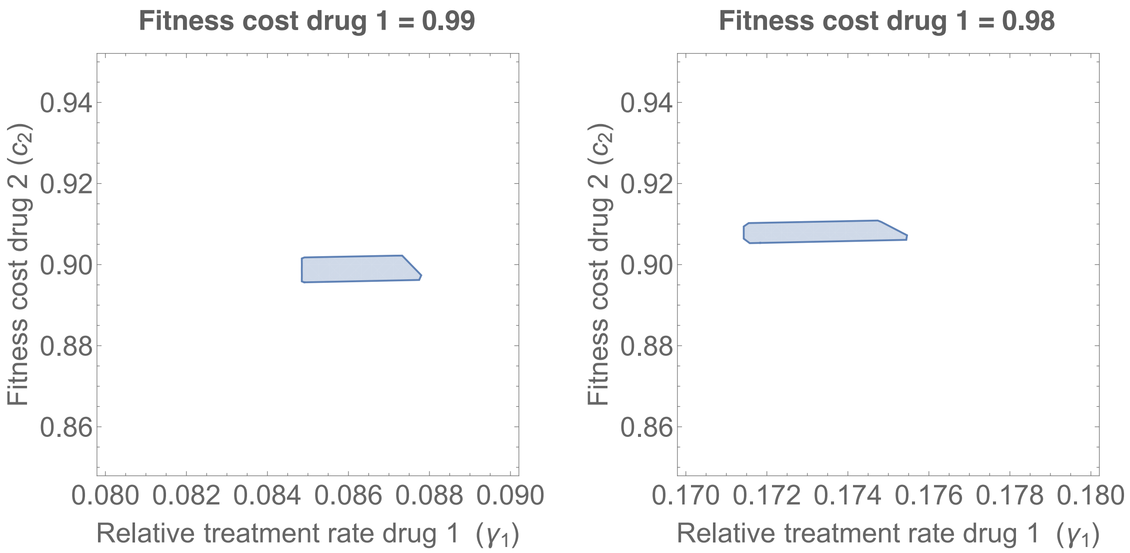

Figure B: The effect of relative treatment rate (γ_1) and fitness cost on whether incomplete linkage disequilibrium is observed. The plots assume one stratum with clearance rate $\mu = 0.65$ and another with clearance rate $\mu = 0.70$ and the shaded area represents where RS is selected for in one stratum and SR in the other. Other parameter values are $\tau = 1/12$, $c_{\beta 1} = c_{\beta 2} = 1$ and therefore $c_1 = c_{\mu 1}$ and $c_2 = c_{\mu 2}$. Small changes in the clearance rates of the strata (e.g. to $\mu = 0.60$ and $\mu = 0.65$) lead to complete linkage disequilibrium being observed for all values of γ_1 and c_2. The same is true for increasing the fitness cost of the less commonly prescribed drug (e.g. $c_1 = 0.97$).

$c_{\mu 2} = 0.9$, $\gamma_1 = 0.09$, $c_{\beta 1} = c_{\beta 2} = 1$ and total antibiotic consumption rate τ of one prescription per year per person, incomplete linkage equilibrium will be observed only if there is at least one strain with clearance rate μ between 0.677 and 0.743 per month and another with clearance rate between 0.668 and 0.677 per month - or, equivalently, duration of carriage between 1.35 and 1.48 months and duration of carriage between 1.48 and 1.50 months (mean duration of carriage is $\frac{1}{\mu}$). If these conditions are not met, incomplete linkage disequilibrium, though theoretically possible, will not be observed.

SI Figure B and SI Figure C further illustrate this sensitivity to parametrisation. In particular, the closer the rate at which the two drugs are consumed, the more restricted the parameter space in which incomplete linkage disequilibrium can arise.

Finally, it is worth noting that when the fitness cost of resistance affects transmission rate only, a similar analysis shows that incomplete linkage disequilibrium never arises. In summary, under specific conditions (the cost of resistance affecting clearance rate and more commonly used antibiotics having a greater fitness cost), incomplete linkage disequilibrium is theoretically possible. However, the narrowness of the parameter range in which this occurs suggests this mechanism leading to incomplete linkage disequilibrium and non-nested resistance profiles being observed is very rare.

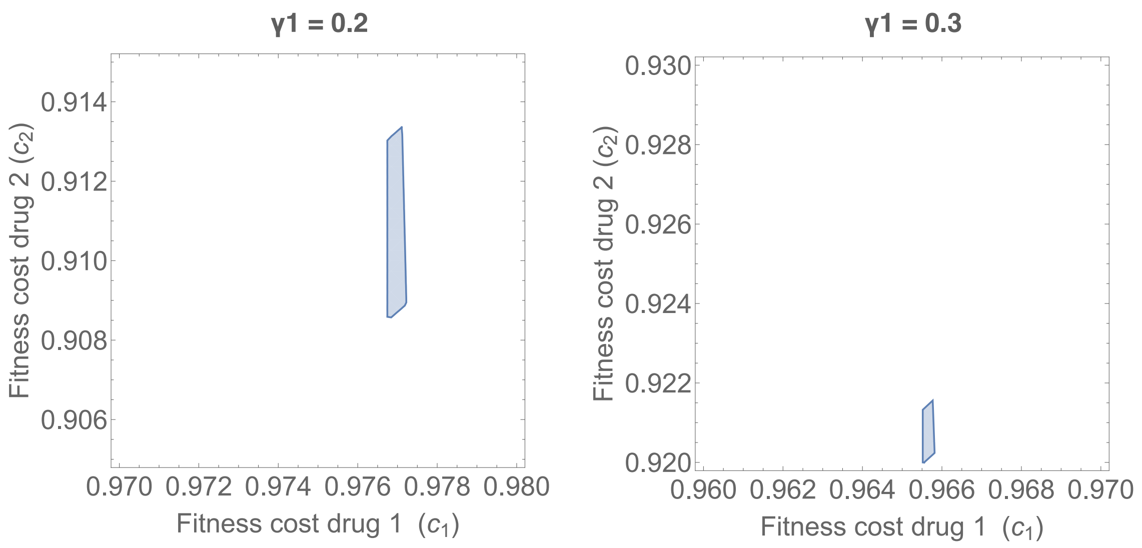

Figure C: The effect of relative treatment rate (γ_1) on the range of fitness costs at which incomplete linkage disequilibrium is observed. The plots assume one stratum with clearance rate $\mu = 0.65$ and another with clearance rate $\mu = 0.70$ and the shaded area represents where RS is selected for in one stratum and SR in the other. Other parameter values are $\tau = 1/12$, $c_{\beta 1} = c_{\beta 2} = 1$ and therefore $c_1 = c_{\mu 1}$ and $c_2 = c_{\mu 2}$. At higher values of γ_1 (e.g. $\gamma_1 = 0.4$), complete linkage disequilbrium in observed for all values of c_1 and c_2.

4 Recombination rate

Allowing recombination at the resistance loci breaks down linkage disequilibrium and therefore decreases the association between resistance determinants: increasing the recombination rate parameter (r) in the model represented by Equation 10 in the main text decreases mean linkage disequilibrium (Figure 3 in main text). The r parameter is not directly interpretable as the frequency at which recombinant strains are transmitted: we assume recombination requires co-infection - how often a host infected by any particular strain transmits a recombinant strain therefore also depends on the frequency of other strains. Here, to make values of r more interpretable, we explore its relationship to the proportion of transmission events involving a recombinant strain: $r \sum_x \sum_y I_{xy}$. (Note that this includes all recombination events, even those leading to transmission of an identical genotype).

Mostowy et al. have estimated that the PMEN1 pneumococcal lineage undergoes recombination events (*at any locus*) at a rate of approximately 0.01 events per month [2]. Pneumococcal transmission rates, on the other hand, have been estimated to be around 1.3-4.3 per month [3], suggesting that transmission of recombinant strains, where recombination has occurred at *any* locus, accounts for 0.2 - 0.8% of transmission. The upper bound of the r parameter range explored in the main text ($r = 0.01$), on the other hand, corresponds to transmission of strains where recombination has occurred *specifically at one of the three resistance loci* making up 1.7% of transmission events (SI Figure D). Some resistance loci in the pneumococcus are recombination hotspots, but even so, this recombination rate is unrealistically high. Values of linkage disequilibrium remain relatively high at this unrealistic rate of recombination (Figure 3 in main text).

It is worth noting, however, that recombination rates for mobile genetic element (plasmid or transposon) associated resistance determinants may be higher if they occur on different elements. Very high rates of recombination would also abolish coexistence - and this effect occurs more rapidly than the break down of linkage disequilibrium (SI Figure E). Therefore, when fitness variation in the effect of resistance acts as a mechanism of coexistence, it will also produce linkage disequilibrium, even in the presence of recombination.

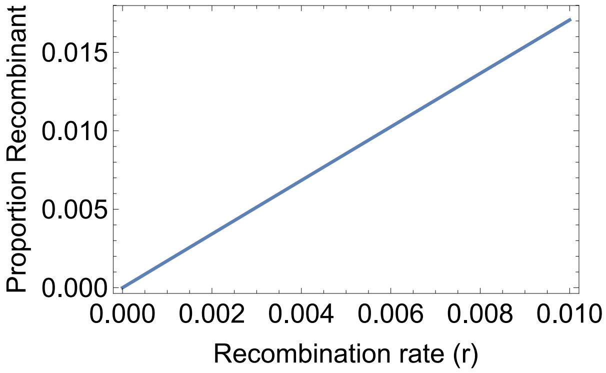

Figure D: The proportion of transmission of recombinant strains as a function of the recombination rate parameter (r) in the model represented by Equation 10 in the main text.

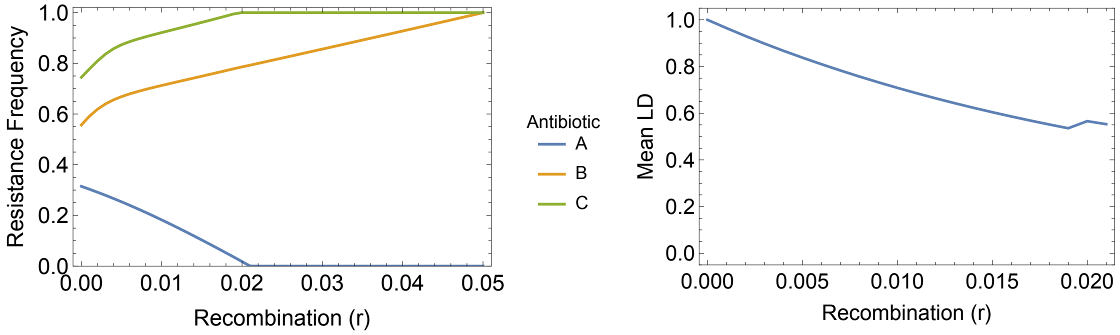

Figure E: Strain frequencies and mean linkage disequilibrium (LD) between resistances in a model with three antibiotics (A, B and C) consumed at different rates and five strains differing in duration of carriage with increasing rates of recombination at the resistance loci (see Methods in main text). Very high rates of recombination eliminate coexistence before the association between resistance determinants. Note the difference in x-axis range: LD is only defined between loci with more than one allele so once coexistence is only observed for a single locus, mean LD can no longer be calculated.

5 Distribution of drug consumption within a stratum

In the multidrug model introduced in Section 2.1.1 (Equation 3), individuals are exposed to a single antibiotic at a time: the total antibiotic consumption rate is the sum of the consumption rates for individual antibiotics ($\sum_j \tau_j$). At low antibiotic consumption rates, this is approximately equivalent to the assumption that different antibiotics are prescribed entirely independently (if $\tau_1$and τ_2 are small, the probability of being prescribed both antibiotics simultaneously ($\tau_1\tau_2$) is very small). However, as discussed in main text Table 1, the assumption of independent prescription is not realistic: aspects of clinical practice lead to correlated antibiotic exposure at the individual level. Conceptually, individual-level correlations can arise in two ways: either because sub-groups within the population have different antibiotic consumption profiles or because antibiotics are sometimes prescribed together as combination therapy. In this section, we explore the effect of modelling individual level correlations in antibiotic exposure within a stratum.

5.1 Sub-groups with different consumption profiles

The presence of sub-groups with difference antibiotic consumption profiles within a homogeneously mixing population does not affect resistance dynamics. To demonstrate this, we consider a population consisting of n sub-groups (note that these sub-groups do not constitute separate strata because these groups are not assortatively mixing). Overall in the population, antibiotics 1 and 2 are consumed at rates τ_1 and τ_2, with sub-group n making up proportions $p_{n,1}$ and $p_{n,2}$ of the consumption of antibiotics 1 and 2 respectively (with $\sum_i^n p_{i,1} = 1$ and $\sum_i^n p_{i,2} = 1$). In a scenario with two sub-groups (a and b) for example, $p_{a,1} = 1$ and $p_{b,2} = 1$ would correspond to group a consuming antibiotic 1 only and group b consuming antibiotic 2 only; while $p_{a,1} = 1$ and $p_{a,2} = 1$ would correspond to group a consuming both antibiotics and group b consuming no antibiotics. With $I_{SS} = \sum_i^n I_{i,SS}$, the dynamics of strain SS in subgroup a are described by:

$$\frac{dI_{a,SS}}{dt} = \beta I_{SS} U_a - (p_{a,1}\tau_1 + p_{a,2}\tau_2 + \mu) I_{a,SS} \tag{3}$$

where β is the transmission rate and U_a are uninfected hosts in sub-group a. As $U = \sum_i^n U_i$, the overall dynamics of strain SS are described by:

$$\frac{dI_{SS}}{dt} = \beta I_{SS} U - (\tau_1 + \tau_2 + \mu) I_{SS} \tag{4}$$

thus recovering the equation from Section 2.1.1 of the main text (Equation 3). The same reasoning applies to all strains. Therefore, the model formulation presented in the main text captures any distribution of antibiotic consumption within a homogeneously mixing population, excluding combination therapy.

5.2 Combination therapy

The model formulation in the main text assumes antibiotics are always consumed separately. To investigate the effect of this assumption, we consider a two drug model which includes simultaneous exposure to both drugs (i.e. combination therapy). In this model, a proportion p ($0 \le p \le 1$) of the drug with the lower rate of consumption (labelled drug 1, without loss of generality) is given in combination with the drug with the higher prescription rate (labelled drug 2). The consumption rate of drug 1 by itself is therefore $(1-p)\tau_1$, the consumption rate of drug 2 is $\tau_2 - p\tau_1$ and the consumption rate of combination therapy is $p\tau_1$. The SS strain is cleared by all three forms of treatment, the SR strain by drug 1 by itself and combination therapy, the RS strain by drug 2 and combination therapy and the RR strain is cleared by no form of treatment. The total clearance rate experienced by each strain (λ_k in main text Equation 3) is therefore:

$$\begin{aligned} \lambda_{SS} = (1-p)\tau_1 + \tau_2 - p\tau_1 + p\tau_1 + \mu = (1-p)\tau_1 + \tau_2 + \mu \\ \lambda_{SR} = (1-p)\tau_1 + p\tau_1 + \frac{\mu}{c_{\mu 2}} = \tau_1 + \frac{\mu}{c_{\mu 2}} \\ \lambda_{RS} = \tau_2 - p\tau_1 + p\tau_1 + \frac{\mu}{c_{\mu 1}} = \tau_2 + \frac{\mu}{c_{\mu 1}} \\ \lambda_{RR} = \frac{c_{\mu 1}}{c_{\mu 2}} \end{aligned} \tag{5}$$

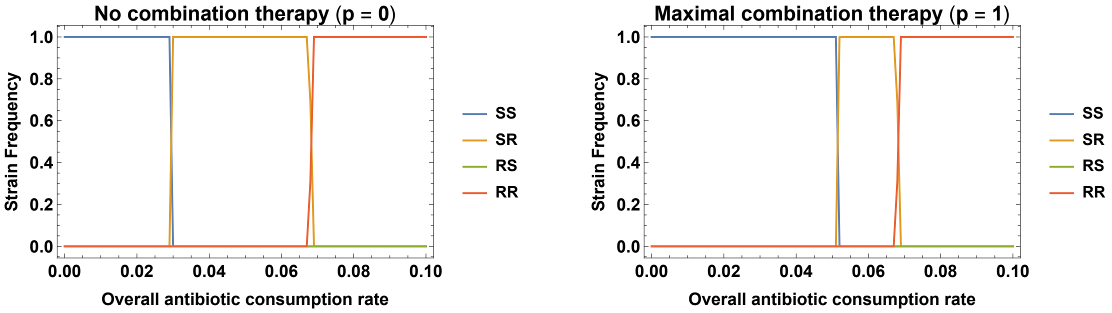

Figure F: The effect of simultaneous antibiotic prescription (combination therapy) on resistance dynamics among a homogeneously mixing population. Combination therapy increases the antibiotic consumption rate at which resistance to the antibiotic with the higher consumption rate becomes beneficial, but does not affect the total antibiotic consumption rate at which multidrug resistance becomes selected for. Total antibiotic consumption (x-axis) refers to the overall antibiotic consumption rate in the population ($\tau_1 + \tau_2$). τ_1 always makes up 30% of the total antibiotic consumption. Other parameters for these simulations are: $\beta = 2, \mu = 1, c_{\mu 1} = c_{\mu 2} = 1, c_{\beta 1} = c_{\beta 2} = 0.98$.

This model can therefore be expressed in the same general form as the multidrug model in the main text (Equation 3) - the result about competitive exclusion therefore also applies when antibiotics are prescribed simultaneously. Furthermore, the proportion of antibiotics administered as combination therapy (p) only affects the clearance rate of the SS strain (λ_k). Inclusion of combination therapy therefore affects the competition between the SR and the SS strain, but not the SR and the RR strain. This is illustrated in Figure F.

Linkage disequilibrium is not defined when allele frequencies are either 0% or 100%, it is therefore not meaningful to discuss MDR over-representation in absence of coexistence. In this model, combination therapy therefore does not promote MDR over-representation within stratum. If within stratum coexistence arises through some other mechanism however, combination therapy might well contribute to promoting linkage disequilibrium within strata.

6 Additional Figures

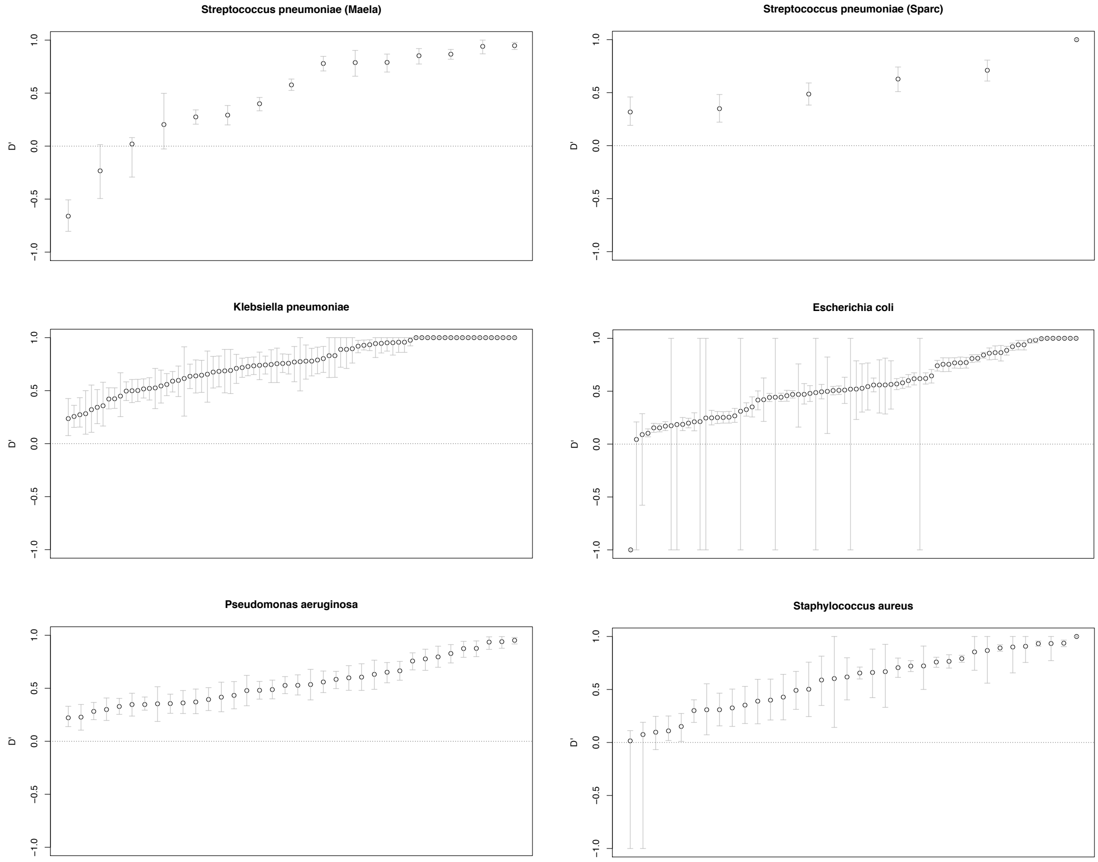

Figure G: Linkage disequilibrium (D') in six bacterial datasets. Each data point represents D' between resistance to two antibiotics, error bars represent 95% confidence intervals (generated by bootstrapping; 1000 bootstrap iterations).

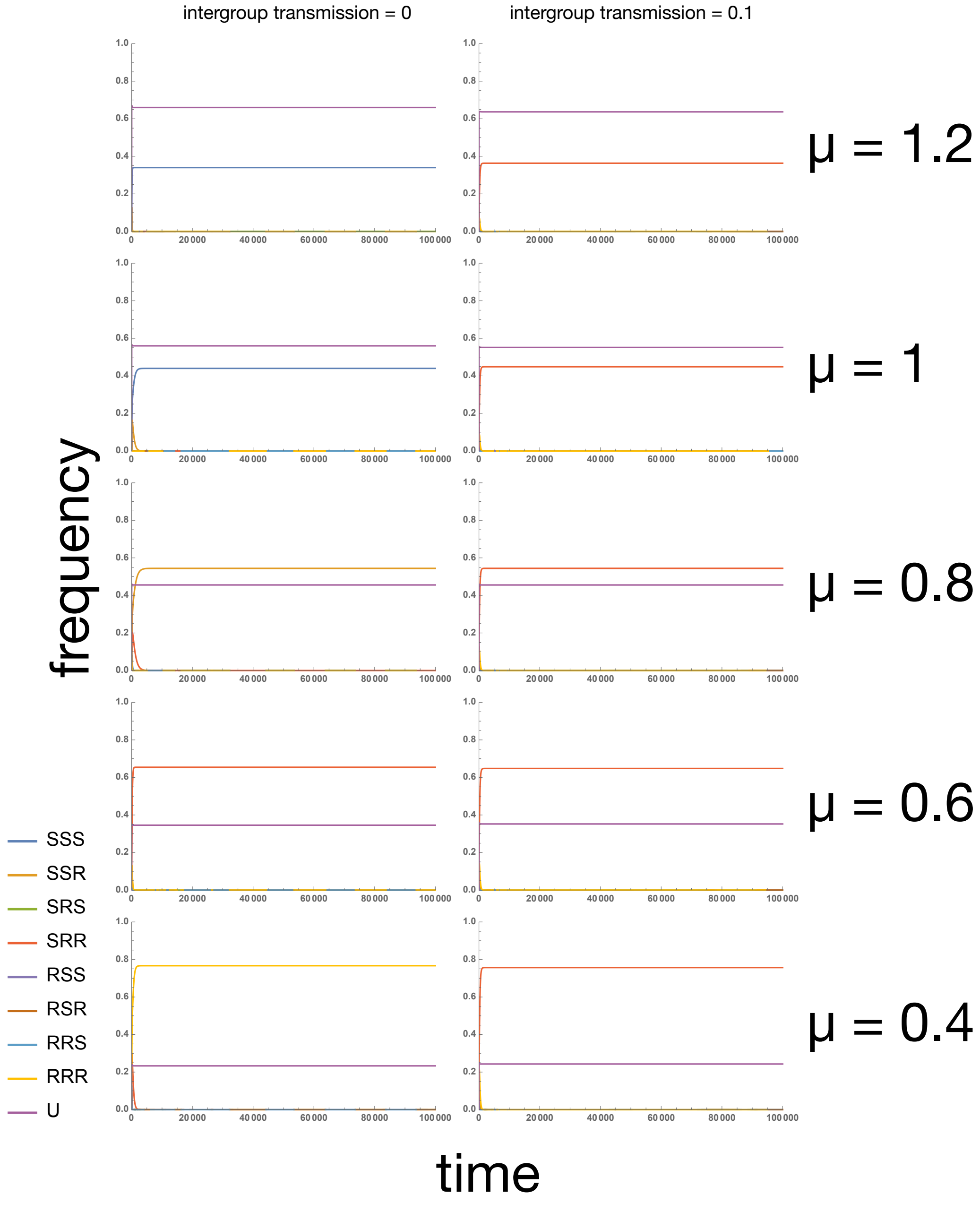

Figure H: Time series of the frequency of infected and uninfected hosts showing that frequencies stabilise before t = 100000 (which is reported as equilibrium frequency in the main text). These plots correspond to the intergroup transmission model (main text Figure 3), with no intergroup transmission in the left column ($m = 0$) and $m = 0.1$ in the right column and other parameters as reported in the main text (see main text Methods). U signifies uninfected hosts, the three letter codes signify the resistance profile (antibiogram) of the strain infecting the host. The model was initialised with 1/8 of the hosts infected with each of the possible strains (as in the figures in the main text).

References

[1] Lehtinen S, Blanquart F, Croucher NJ, Turner P, Lipsitch M, Fraser C. Evolution of antibiotic resistance is linked to any genetic mechanism affecting bacterial duration of carriage. Proceedings of the National Academy of Sciences of the United States of America. 2017;114(5):1075–1080. doi:10.1073/pnas.1617849114.

[2] Mostowy R, Croucher NJ, Hanage WP, Harris SR, Bentley S, Fraser C. Heterogeneity in the frequency and characteristics of homologous recombination in pneumococcal evolution. PLOS Genetics. 2014;10(5):e1004300.

[3] Colijn C, Cohen T, Fraser C, Hanage W, Goldstein E, Givon-Lavi N, et al. What is the mechanism for persistent coexistence of drug-susceptible and drug-resistant strains of Streptococcus pneumoniae? Journal of The Royal Society Interface. 2010;7(47).
